# Selfie consents, remote rapport, and Zoom debriefings: collecting qualitative data amid a pandemic in four resource-constrained settings

**DOI:** 10.1136/bmjgh-2020-004193

**Published:** 2021-01-07

**Authors:** Mark Donald C Reñosa, Chanda Mwamba, Ankita Meghani, Nora S West, Shreya Hariyani, William Ddaaki, Anjali Sharma, Laura K Beres, Shannon McMahon

**Affiliations:** 1 Heidelberg Institute of Global Health, Ruprechts-Karls-Universität Heidelberg, Heidelberg, Germany; 2 Department of Epidemiology and Biostatistics, Research Institute for Tropical Medicine, Department of Health, Manila, Philippines; 3 On behalf of the Social & Behavioural Science Group, Centre for Infectious Disease Research in Zambia, Lusaka, Zambia; 4 Department of International Health, Johns Hopkins University Bloomberg School of Public Health, Baltimore, Maryland, USA; 5 Johns Hopkins India Private Limited (JHIPL), Delhi, India; 6 On behalf of the Social & Behavioral Sciences Team, The Rakai Health Sciences Program, Rakai, Uganda

**Keywords:** qualitative study, public health, health policies and all other topics, study design

## Abstract

In-person interactions have traditionally been the gold standard for qualitative data collection. The COVID-19 pandemic required researchers to consider if remote data collection can meet research objectives, while retaining the same level of data quality and participant protections. We use four case studies from the Philippines, Zambia, India and Uganda to assess the challenges and opportunities of remote data collection during COVID-19. We present lessons learned that may inform practice in similar settings, as well as reflections for the field of qualitative inquiry in the post-COVID-19 era. Key challenges and strategies to overcome them included the need for adapted researcher training in the use of technologies and consent procedures, preparation for abbreviated interviews due to connectivity concerns, and the adoption of regular researcher debriefings. Participant outreach to allay suspicions ranged from communicating study information through multiple channels to highlighting associations with local institutions to boost credibility. Interviews were largely successful, and contained a meaningful level of depth, nuance and conviction that allowed teams to meet study objectives. Rapport still benefitted from conventional interviewer skills, including attentiveness and fluency with interview guides. While differently abled populations may encounter different barriers, the included case studies, which varied in geography and aims, all experienced more rapid recruitment and robust enrollment. Reduced in-person travel lowered interview costs and increased participation among groups who may not have otherwise attended. In our view, remote data collection is not a replacement for in-person endeavours, but a highly beneficial complement. It may increase accessibility and equity in participant contributions and lower costs, while maintaining rich data collection in multiple study target populations and settings.

**Summary box**

- Qualitative researchers have historically championed gathering respondents’ perspectives via face-to-face engagement, but the ongoing pandemic presents challenges to in-person research.
- In shifting to remote data collection—via mobile phones or online formats—we identified challenges related to rapport building, fear of technology, privacy and confidentiality, and developed measures to address this.
- Drawing from our qualitative data collection experiences in the Philippines, Zambia, India and Uganda, this paper outlines exemplars, mitigation techniques and lessons learned that could inform remote interviewing strategies beyond the COVID-19 context.

## Introduction

As qualitative researchers, we champion the value and necessity of rapport building, empathy, open and honest dialogue, and a sense of closeness between research teams and interview respondents. Throughout our careers, we have adhered to a longstanding (if unstated) view that face-to-face engagement, in a location that is comfortable for and familiar to the respondent, is the gold standard in qualitative data collection—and anything else is second best.^1 2^ Face-to-face interviewing facilitates a qualitative researcher’s ability to observe non-verbal cues (eg, furtive glances, fidgeting, or an eye roll), use silence as an element of patient dialogue, and to record and probe about the artefacts or tools that reflect a person’s life (eg, the material objects that hold meaning or value for an individual).^3^ COVID-19 and associated lockdowns and social distancing have forced us to challenge these perceptions in pursuit of gathering trustworthy, rigorous and authentic qualitative data in low- and middle-income countries (LMICs).^4–6^

Several academics, often doctoral students, have highlighted the pros and cons of collecting data remotely.^7–9^ James and Busher described doctoral data collection using email, and noted disadvantages of the asynchronous approach, which could sometimes cause a loss of coherence and flow of thought, leaving the data feeling ‘dry’ due to an absence of visual and auditory cues.^9^ The authors also highlighted concerns about consent and anonymity given the nature of electronic messaging and data storage.^9^ Similarly, researchers using phone interviews to collect qualitative data described a lack of non-verbal data, which contributed to a limited understanding of context.^10^ Several others, however, detailed the benefits of phone interviews offering richer discussions on sensitive topics due to increased perceptions of anonymity,^11 12^ and improved access to hard-to-reach respondents^13^ and settings that may otherwise be considered unsafe for research.^14^

More recently, studies have examined video communication platforms such as Zoom, Skype or WhatsApp,^8 15–18^ and identified mixed, but largely positive experiences. Deakin and Wakefield highlighted tremendous potential for Skype to facilitate data collection across a wide range of geographical perspectives while operating on modest budgets.^15^ At least two studies directly compared in-person to online communication,^8 16^ and found relatively modest differences across the approaches in terms of participant satisfaction and data quality,^8^ although microphones, webcams and uneven internet reliability presented challenges. Most recently, studies have explored the use of mobile instant messaging applications to elicit respondents' daily experiences, feelings and thoughts.^17 18^ Kaufmann and Peil^18^ state that the use of WhatsApp messaging has proven useful in capturing participant’s daily experiences via multimedia options including pictures, videos, screenshots, emojis, filters and hashtags.

A majority of literature on the use of remote means (eg, internet or phone based) to gather qualitative data precedes the current COVID-19 pandemic, and comes from high-income countries (HICs). As noted above, researchers working in HICs have highlighted that remote data collection facilitates reaching people who are isolated, geographically dispersed, stigmatized, overlooked or ignored.^19–22^ They note the novelty of remote data collection, because it represents a substantive adaptation or pivot from the status quo. In contrast, there is little research on remote data collection in LMICs. A counterpoint to expanded participation, remote data collection may create or foment selection bias because access to electricity, mobile phones, and the Internet, while expanding, is not nearly as universal in LMICs as in HICs.^23–25^ Though mobile phone ownership among women has been increasing, a gender gap persists: women are 10% less likely than men to own mobile phones across LMICs with the largest gap observed in South Asia.^23^ Similarly, women in LMICs are 23% less likely than men to use ‘mobile internet’, a term that refers to accessing the internet via a smartphone or tablet using a wireless or cellular connection.^25 26^ Broadly speaking, rural populations in LMICs are also 40% less likely to use mobile internet than urban populations.^25^ Hence, while researchers in LMICs have had to adapt and pivot for decades in the interest of getting data amid major structural challenges (we have, for example, contended with natural calamities, political unrest, epidemics and resource shortages), we have rarely considered electronic or mobile data collection as a promising solution.

In relation to the current pandemic, we are aware of blog entries^27^ and Twitter discussions, though relatively little academic literature to guide the research community, particularly the qualitative community, on how to adapt amid the ongoing pandemic. In this practice paper, drawing from our experiences collecting data remotely via online and mobile phone-based interviews across four LMICs, we share methodological and practical adaptations and lessons learned to guide fellow qualitative researchers who are contending with the ongoing pandemic—and who may want to consider remote means of data collection well into the future. We do not emphasize general tenets of qualitative research, or tips for collecting high-quality qualitative data generally, but instead focus on remote qualitative research specifically.

## Case studies

Our case studies stem from research underway in the Philippines, Zambia, India and Uganda. While comprehensively discussing comparative historical, cultural, structural and social differences is beyond the scope of this paper, we present a snapshot of demographics, COVID-19-related details, pertinent information regarding each country’s access to electricity, mobile phone subscriptions, internet connectivity and information related to our ongoing research (table 1).

**Table 1:** Country characteristics

	Philippines	Zambia	India	Uganda
**Covid-19 related**				
First confirmed case*	January 30, 2020	March 18, 2020	January 30, 2020	March 21, 2020
Total confirmed cases as of October 5 2020*	322 497	15 052	6 623 815	8808
Total deaths as of October 5 to 2020*	5776	333	102 685	81
Deaths per 100 000 population†	5.3	1.8	7.4	0.2
**Electricity, internet, phone**				
Access to electricity (% of population)‡	95 (total) 98 (urban) 93 (rural)	40 (total) 77 (urban) 11 (rural)	95 (total) 100 (urban) 93 (rural)	43 (total) 58 (urban) 38 (rural)
Mobile§ subscriptions per 100 people	154	96	84	57
Secure Internet servers per 1 million people¶	111	36	389	22
Individuals using the Internet (%)**	43	14	20	24
**Population characteristics**				
Population††	108 million	18 million	1.4 billion	44 million
Average age (median)‡‡	25.7	17.6	28.4	16.7
**Descriptions of our qualitative research**				
Topical focus	Vaccine hesitancy	TB care-seeking	COVID-19 health services	Mental health and HIV
Target population	Parents of <5 children, policy makers, healthcare workers, community leaders	Patients diagnosed with TB in the 2 weeks prior to interview	Private healthcare providers, including medical doctors and experience-based rural medical practitioners who provide services to low-income populations	People living with HIV, health workers, community members knowledgeable about mental health
Geographical areas	Urban and rural	Urban	Urban and rural	Rural, trading and fishing communities
Sampling technique	Purposive—criterion	Purposive—criterion	Purposive—criterion and snowball	Purposive—criterion and snowball
Average length of interview	1–1.5 hours	45–60 min	45–60 min	1–2 hours
Remote platforms used for recruitment and/or interviews	Zoom, Skype, Google Meet and FB messenger	Mobile phone	Mobile phone, WhatsApp phone, Zoom	Telephone

*https://covid19.who.int.

†https://coronavirus.jhu.edu/data/mortality.

‡https://data.worldbank.org/indicator/EG.ELC.ACCS.ZS.

§https://data.worldbank.org/indicator/IT.CEL.SETS.P2?name_desc=true.

¶https://data.worldbank.org/indicator/IT.NET.SECR.P6.

**https://data.worldbank.org/indicator/IT.NET.USER.ZS.

††https://data.worldbank.org/indicator/SP.POP.TOTL.

‡‡https://apps.who.int/gho/data/node.imr.WHS9_88?lang=en.

TB, tuberculosis.

We begin by highlighting our experiences in the field and the challenges both prior to and during data collection with special emphasis on an overarching theme or challenge that emerged within a given research team, and the workaround pursued to mitigate this challenge.

### Case study 1: overcoming fear of online interviewing in the Philippines

Fear is perhaps the best word to describe our collective feeling upon realizing that an online shift was inevitable in order to collect data for ‘Project SALUBONG: Building Vaccine Confidence via Empathy and Narratives’ in the Philippines. We feared how review boards, fellow scientists and research participants would react, particularly because vaccines are a controversial topic, and we felt that controversial topics necessitate direct, in-person engagement. Fear also describes the perspective of our interview teams in terms of engaging with online platforms. Several of our younger data collectors are tech-savvy, and highly conversant on the nuances of tech and ‘tech speak’; they understand toggling, and amplify their communication styles with hashtags and emojis. Meanwhile, many of our older staff members are self-proclaimed ‘technophobes’ who felt overwhelmed by the number of buttons and navigation links on mobile devices and computers. We addressed these fears head-on. We modified trainings to include modules on computer applications, video calling platforms and online voice recorders, as well as data backup and protection procedures. To train interviewers, we used Zoom breakout rooms, which allowed interviewers to practice interviewing techniques in different groups, with and without supervision from trainers, but we always ensured that a tech supporter was ready to support any tech-related snafus. We practiced recruiting, consenting and interviewing online, including modules on ‘tech disruptions’ so that research assistants would have to develop workarounds if a screen froze or a call dropped. We also developed a phone script to facilitate the recruitment process (see online supplemental file 1) and trained our techno-reticent researchers on multiple platforms that participants described preferring (eg, Facebook messenger, Zoom, Google Meet or Skype). For consenting, in lieu of meeting participants in person and establishing informed consent by signature or fingerprint, participants signed consent forms remotely during a recorded video call, and shared a ‘selfie’ with the signed form. To ensure participants’ internet connectivity throughout the interview, we purchased and transmitted free mobile data packages in advance. Lastly, to bolster transnational collaboration amid travel restrictions, we conducted systematic debriefings via Zoom at the end of each day of data collection to share experiences and improve study procedures.^28^

10.1136/bmjgh-2020-004193.supp1 Supplementary data

### Case study 2: allaying respondent suspicions and building mobile rapport in Zambia

Our study sought to understand care-seeking experiences and preferences among newly diagnosed (<3 weeks), adult patients with tuberculosis (TB) at three health facilities, identified through health facility registers. We transitioned from the planned in-person to mobile phone-based data collection. When calling potential participants, we first confirmed the identity of the person answering the phone by asking for details that we could verify via facility-based client records, such as their name and recent care-seeking behaviour. Persons called were often suspicious, questioning how and why they were contacted. Providing a clear and comfortable introduction was thus part of rapport building, requiring interviewers to allay concerns by quickly outlining our purpose and explaining how we obtained their phone number. Mentioning their health facility in the introduction ‘signaled’ the interview topic, leading some to immediately decline participation. For others, the association with the health facility built trust and credibility, including allowing participants to confirm the study’s aim with facility staff prior to participation. Additional rapport-building followed usual in-person techniques of answering participant questions, listening carefully, starting with comfortable topics, and using third-person examples for sensitive questions. We had thought phone interviews might be shorter, or that data gathered by phone may be less forthright or revealing. In fact, this was not the case. In comparison to in-person in-depth interviews (IDIs), participants’ tone of voice and the detailed narration of their experiences suggested that, for many respondents, it was easier to discuss sensitive topics and challenging life experiences while *not* in the physical presence of another person. Rapport extended beyond the initial interview, with several participants seeking TB or COVID-19 information from researchers during or after the call (in order to provide consistent information, we created COVID-19 interviewer scripts that included referral phone numbers). To prevent possible problems, early in the interview we discussed data use and/or times for a follow-up call in case of an abbreviated interview due to network or phone battery challenges, and we collected details required for mobile money reimbursement. Regular research team debriefs over Zoom and memos written within 24–48 hours post interview helped us to address challenges in real time.

### Case study 3: rapid recruitment of respondents for remote interviews in India

Our study aims to provide immediate, actionable evidence to inform the government’s efforts on leveraging the private health sector’s capacity to meet the health needs of poor and vulnerable populations, like migrants, who have been disproportionately affected by COVID-19 in Uttar Pradesh (UP), India. Given the diversity of private health providers who play a critical role in providing services to these populations—ranging from small nursing homes and single-doctor clinics to experience-based practitioners, such as rural medical practitioners (RMPs)—we have had to adopt different strategies to remotely recruit respondents for phone and online interviews during the pandemic. First, we identified professional networks of private health providers (eg, allopathic, Ayurveda, Yoga & Naturopathy, Unani, Siddha and Homoeopathy), and experience-based practitioners at state and district levels. Building rapport with the Heads of health associations and district health leadership over multiple phone conversations and engaging them as key informants proved to be a useful strategy to recruit both providers from small hospitals and nursing homes as well as experience-based practitioners across the study sites in UP. We complemented this strategy by identifying other small hospitals and larger hospitals through UP’s Health Management Information System and cold calling them using a recruitment script that was designed to introduce the research objectives as well as establish researcher and institutional identity. We found our institutional affiliation with Johns Hopkins University brought legitimacy to our interactions with respondents who we had directly approached. Lastly, we relied on snowball sampling as an important recruitment strategy and found it to be especially effective for identifying single-doctor clinicians, as well as, gaining their trust in interviews. In addition, snowball sampling was particularly important for reaching RMPs, given our inability to conduct an in-person mapping exercise to identify them. Overall, conducting remote interviews has allowed for an unexpected level of speed and flexibility with scheduling. Often our respondents have been willing to participate in a phone interview on the same day or the next, and they have been willing to schedule interviews outside normal working hours, for example, during evenings and weekends. Furthermore, with data collectors based across time zones, we have had a unique opportunity to schedule interviews during early mornings, afternoons and late evenings, per the respondents’ convenience.

### Case study 4: addressing interview fatigue in Uganda

‘Musawo [health care worker], these questions are many’. This statement was featured in one of our first in-person interviews, conducted prior to the national lockdown that halted data collection. Interviews were running well over an hour, and some participants seemed impatient by the end, with responses becoming thin. Our study uses a variety of qualitative methods to engage participants on the often difficult-to-discuss topic of mental health among people living with HIV in South-western Uganda. As we navigated shifting to telephone-based data collection, we were particularly concerned about fatigue and patience based on experiences in prior interviews. Surely participants would be more likely to get fatigued, impatient, and distracted when over the phone, and now we would not be able to see it. We shortened our guides, but wondered if it was enough. We had also lost our ability to use a timeline visual that we had developed. It had centred the interviews and worked well. It was now condensed into a script—more added time! To address these concerns, interviewers developed strategies for explaining the timeline by first summarising the points on the timeline and stating they would walk through time points in chronological order. Interviewers continued to keep a hardcopy of the timeline in front of them during the interview, allowing the tool to guide questions. We discussed plans in case participants wanted to cut interviews short or seemed tired, such as having a pre-agreed on back-up time, and considered if we should split the interviews into two sessions. When recruiting participants, we stressed they should find a comfortable and private place for the interview. To build rapport, we chatted briefly about the rainy season, well-being of their family and checked-in verbally throughout interviews: ‘Are you still doing ok?’, ‘Is the time alright for you?’. To our surprise, interviews ran over an hour but participants were not fatigued, with rich responses continuing through to the end of the interviews. Only one person has refused participation to date.

## Adapted qualitative components amid the pandemic

The continuing need for qualitative interviewing to personalize and adapt during the pandemic suggests unlearning and re-learning some of the traditional approaches that have shaped the discipline. In table 2, we break down the deceptively ‘simple’ act of remote interviewing across all of our case study settings and by study phases (from training data collection teams to conducting debriefings post-interviews), using succinct bullet points to guide qualitative research teams as they collect data remotely.

**Table 2:** Challenges, mitigations and lessons learned amid remote, qualitative data collection across four settings

Challenges exacerbated by remote approaches	How we mitigated these in our studies	Lessons learned
**Research phase: data collector training**		
All research team members need to know how to use the remote technology: Internet, break-out room creation, etc. Greater need to train research team members on how to navigate and prioritize sections of interview guides to allow for unplanned, abbreviated dialogues (if electricity or internet drops) and to reduce silences that aren't linked to probing (reducing the time that an interviewer might shuffle through papers) Active listening, concentration, and attentiveness become even more important when language is the only tool to communicate	Do special, opt-in pretraining on online learning prior to the start of formal training for those who are less tech savvy Embrace (and openly recognize) that some team members have strengths that others lack; urge openness and patience with tech challenges Facilitate interviewer familiarity and practice with interview guides in advance of implementation Spend time revisiting interviewing techniques	More time is needed for staff to practice using online platforms and to pilot interview guides Build an “experiential” practice team to create a win-win situation (a dyad of a low-tech and high-tech person) Have a stand-by ‘go-to’ person to help troubleshoot concerns (someone from information technology or someone who is relatively more tech-capable in the team) Provide timely and targeted feedback on remote interviewing techniques during practice or pilot interviews, even for experienced interviewers
**Research phase: respondent recruitment**		
Getting permission from local authorities and regulatory bodies Identifying and electronically coordinating with gatekeepers (healthcare providers or village health teams for facility-based recruitment; community groups, and community health workers for neighbourhood recruitment; city councils for established community groups; state or district health/medical associations for private health providers) National regulations on use of communication technology and use of phone numbers to reach a wider audience Participants may not be prepared or feel comfortable to undertake interviews at the time of recruitment, particularly when cold called. They may feel suspicious about how you got their contact information and why you are contacting them	Communicate via multiple, official pathways (email addresses *and* letters via official channels such as couriers *and* phone calls) Develop a phone script for the remote recruitment process to ensure you have reached the right person before inviting them to participate in the study Place special focus on introducing yourself and your organisation as well as explaining how you got the respondent’s phone number. Give a chance to the participant to verify that the person doing recruitment is not an impersonator Allow participants to pick the date and time of the interview and reinforce they should schedule the interview for when they can be in a private, quiet place, and have their phone charged (for phone-based interviews), and can be prepared to write down important contact details (when using verbal consent) Have the same person doing recruitment be a part of data collection, where possible.	Establish a good working relationship with the respective secretaries or focal persons of the local authorities to ensure study follows highest ethical and legal standards Via phone or live video (not recorded to ensure data privacy), partner with gatekeepers and stakeholders in selecting study participants based on your inclusion and exclusion criteria Call potential participants to set-up phone-call meetings; and, any follow-up communication to clarify the research and any pending permissions, review or approvals The recruitment process provides an opportunity to prepare participants for the differences between in-person and remote interviews and to build initial rapport At recruitment, emphasize to participants the need to fully charge phone batteries and or access a reliable phone
**Research phase: consent**		
Getting consents correctly and privately Ensuring ongoing consent both throughout the interview, and at each interaction if conducting longitudinal and iterative interviews	Develop a standard operating procedure to ensure that elements of a good informed consent process can be achieved (e.g. give complete but shortened information followed by 2–3 questions to confirm comprehension) If written consent is required, send copies of information sheet and consent forms days prior to the discussion via email, text message (e.g. WhatsApp, Viber) or courier Use verbal consent procedures and audio-record confirmation of comprehension and consent Provide reminders to respondents to return signed consent forms in advance of interviews by email or text message (e.g. WhatsApp)	Partner with community health workers to help distribute information sheets and consent forms; and collection of signed informed consents Work closely with local ethical boards to ensure that your electronic procedures align with good health and research practices Audio/video record the signing or statement of consent Accept local preferences if they align with local ethics review boards (e.g. in one setting, we found that respondents prefer ‘*selfie*’ consents so we adopted it) If printing, signing and sharing written consent forms is not possible (due to lack of access to printer, fax machine, etc.), consider an electronic/digital signature as an endorsement of consent
**Research phase: preparing for the interview**		
Ensuring a stable internet connection Finding a private and quiet space, conducive for interviews for researchers and participants Building sufficient trust to allow sensitive discussions Ensuring dedicated, charged mobile phones with sufficient talk time for researchers and participants Identifying ways to reimburse participants for their time	Provide free mobile data packages or access to phones to participants and data collectors Empower participants to choose any online platform they like Reinforce that the study can provide technical assistance to participants and data collectors in setting up online platforms to alleviate fear of technology Arrange mobile money set-up to supply remote participant reimbursement	Prepare to invest much more time in this phase as a means to build rapport Exchange more dialogue in whichever preferred mechanism the respondent suggests (emails, WhatsApp, Facebook messenger, phone calls, etc.) Be transparent. Let participants know if someone is with you during the interview session (i.e., presence of a note taker or observer); introduce this person and let the other person say their pleasantries Prepare for additional follow-up to ensure respondents have received reimbursements (e.g. via mobile banking or airtime incentives)
**Research phase: conducting the interview**		
Difficulty following the flow in a natural way due to outside (or technology related) distractions Participant suspicion around why interviewer is calling, who they are and how their number was obtained Needing to confirm to whom interviewer is speaking while avoiding possible disclosure of sensitive information (e.g. HIV status) Participants requesting call-backs at later times when phone available to them or when they are not busy Possibility of phone cutting due to network or charging issues. Being prepared with latest information and national guidance for possible questions related to COVID-19 (even if this is not the focus of the study)	Confirm that participants have private, quiet place to engage in interview Have a clear opening/interview introduction, explaining interviewer identity, how participant’s phone number was obtained and, if possible, association with clinic or institution (as a means to lend credence) Ask for intended participant by name, be prepared to provide a benign reason for calling that can be given if the intended person does not answer the phone so as not to raise suspicions about health issues Clarify among the research team what is allowable for participants based on study protocol and nature of interviews (e.g. can another person be present in the interview) Be flexible in terms of timing (conducting interviews in the morning/evening before respondents begin their workday) Prioritize most important questions first, probe sub-themes if time permits Give data collectors scripts regarding COVID-19 and phone numbers to refer people with additional questions	Invest in good lighting devices to facilitate facial expressions and eye contact when video calling Wear identifying material if that seems appropriate (ID card, etc., to affirm your research role) Wear a headset to minimise background noise Set up downloadable video- and audio recording tools (e.g. Audacity, Movavi Screen Captures, and QuickTime) Practice the recording formats and ways of computer transfer in advance Use parallel audio recorders as back ups Prepare for the reality that many interviews will last the full, planned time period (or even longer) despite being over the phone While not everyone owns a phone, respondents will often guide researchers to a workaround (a phone in a health facility, a shared communal or family phone)
**Research phase: debriefing teams post interview**		
Difficulty gathering all team members since interviews happen at very different times and teams are in disparate locations	Schedule debriefings at a time in the evening rather than immediately after data is collected Practice Zoom—and Zoom breakout rooms—for those who require a refresher Provide a structured debrief form for data collectors to fill out after data collection (and send via email or sync to a shared file) to capture immediate postinterview reflections	Make sure data collectors get into a rhythm in relation to a debriefing timeline For those who are less tech savvy, if they speak less in a debriefing, draw out their insights more pointedly and make sure the tech supporter (see text above) is on hand

## Notable challenge: accessing rural and remote populations

We note that in many settings, rural populations are less likely to have mobile and/or internet access, which facilitated enrollment in our case studies. In Uganda, participants (who are people living with HIV) were drawn from an open, population-based cohort study.^29^ Cohort study participants are asked to provide a telephone number, even if they themselves do not own the phone. Sampling from this existing study with robust procedures in place to obtain contact information increased our ability to reach participants, particularly those in rural areas. Our Uganda-based study is focused on eliciting local models of mental health and although remote data collection may limit the range of perspectives, we feel we are still able to achieve our objectives despite being unable to enroll individuals who lack telephone access. Given the rapid proliferation of mobile technologies, even in rural settings,^30^ strategies beyond cohort designs to engage participants could include multiple recruitment attempts at different times of day and over a period of time to attempt to make contact when someone is in signal range, and/or supporting access through community healthcare workers and others in closer geographical proximity, and/or scheduling contacts for a time when they can share a mobile device. In India, identifying private providers located in rural locations was difficult in the absence of an existing roster of providers. Once we are able to establish contact with 1–2 providers through snowball sampling however, the lack of access to mobile phones or internet connectivity was not a substantial barrier for conducting remote interviews.

## Unanticipated benefits of remote data collection

Beyond challenges, remote data collection presents unforseen benefits and opportunities. These opportunities include direct study benefits (eg, faster recruitment), to broader impacts such as reduced carbon dioxide emissions (table 3).

**Table 3:** Unforeseen opportunities

Video interviewing	Oftentimes, we code transcripts. Verbatim transcripts are an excellent way to tease out verbalized features of a conversation. However, much of the depth of a conversation may be lost because ‘silent’ communication is not captured during transcription. Videos allow us to code not just the text, but also much of the body language and emotional texture of an interview in a manner that may not otherwise be possible.
Recruitment	Recruitment was generally faster, particularly in urban settings or settings with strong internet or mobile access. Additionally, we did not experience a higher refusal rate compared with face-to-face data collection.
Low costs	Face-to-face interviewing requires travel across several locations, some of which may be in hard-to-reach rural areas, requiring high financial costs (ie, vehicle access, fuel, per diems and accommodation of the research team). Remote data collection can be done on a reduced budget.
Minimization of environmental dilemmas	Online and phone-based approaches reduce ecological and carbon footprints because researchers and teams are not travelling to/within countries.
Reduce possibility of awkwardness and embarrassment on sensitive topics	Our data suggest that, for some respondents (and possibly also for some research assistants), discomfort seems to be reduced when using remote formats. Discussion of sensitive information was often much easier remotely.
Skills building	Research assistants appreciated learning how to use remote technologies, representing an added skill that is transferrable to several aspects of their professional and personal lives.
Expanded data collection opportunities	Remote data collection may expand opportunities for participation to individuals who would not have been able to travel to enrol in a study. Additionally, investigators and students who are not based locally have an increased ability to participate in and lead data collection activities.

## Conclusion

We found that conducting qualitative research remotely can initially be daunting, as it requires diverging from common and familiar procedures both prior to and during data collection. Some of our researchers and participants were hesitant—and even technophobic—at the outset of the process. However, with new and adapted procedures, comprehensive training, continuous debriefings to address emerging issues, and increasing familiarity with processes, it was possible to collect high-quality data. Remote data collection allowed broad and rich participation in each of our case studies, proving effective for our populations of interest. We caution, however, that there may be challenges reaching participants in areas where telephone or internet access is poor, requiring inventive strategies to improve enrollment or requiring that researchers be forthright about recruitment limitations. In our view, remote data collection is not wholly a replacement for in-person endeavours, but it is a highly beneficial complement to such approaches. We plan to incorporate online and mobile data collection into our future research efforts, regardless of pandemic-related restrictions.

## Data Availability

No data are available from this practice paper.
