# Supplementary material for: Selfie consents, remote rapport, and Zoom debriefings: collecting qualitative data amid a pandemic in four resource-constrained settings
Source: BMJ Glob Health. 2021 Jan 7;6(1):e004193. doi: 10.1136/bmjgh-2020-004193 (PMC7798410; doi:10.1136/bmjgh-2020-004193)
Supplement: Supplementary data [file bmjgh-2020-004193supp001.pdf]

Supplementary Material

Supplementary File 1. Phone Script for recruitment of participants in the Philippines
(Developed by Mark Donald Reñosa)

Good Morning / Afternoon! I am (*state name of the study staff, and state position*) from (state name of institution and address). I am looking for (*name of the potential participant*).

[ ] Correct person (proceed to the next statements)
[ ] No (ask for the person OR if confirmed to be wrong number, say your apologies and end call)

Ma'am/ Sir, I got your number from (*state name where you get his/her number*) and you are chosen to participate in a study entitled: (state title of the study). Can I have a few minutes of your time to invite you to participate in the study?

[ ] Yes (proceed to the next statements)
[ ] No (ask for the availability for the next call or if not interested, say thank you and end call)

We are inviting ___ participants (*insert number of participants, and specify study population*) to participate in the study from the cities and municipalities (*state study locale*) for an interview. The purpose of this project is to (*state purpose of the study*). The principal investigators are (*state the name of investigators*). Participation in this study is voluntary and free of costs. You have the right – without having to give reasons – not to participate in this study. Should you decide to join or not to join in this activity, it will not affect your standing or your rights to any services in the community.

Should you be interested to participate, we will explain the full study details via online platforms of your choice (Facebook call, Skype, zoom etc.). We will set time and schedule the online discussion to answer questions and concerns and for the signing of the consent forms. Do you want to know more of the study?

[ ] Yes (proceed to the next statements)
[ ] No (say thank you and end the call)

May I know your preferred date and time? Date: ______________________
Time: _

Ma'am/ Sir, we will be sending preliminary copy of the information sheet and consent form to you via online for your further reading before our online discussion and before deciding to participate. Where do you want us to send the preliminary copies of the documents?

[ ] Email (get email address): ________________________________
[ ] Facebook (get Facebook account): ________________________
[ ] Other online platforms: Skype, Viber or WhatsApp (get accounts) ______________________

Further, we will be sending hard copies of the document via a courier. Can you provide us your complete home address?

____

Ma'am/ Sir, we will also provide prepaid loads to assist you with the Internet connection. What video-call online platforms are you comfortable using?

[ ] Skype or Zoom (get accounts): ______________________
[ ] Facebook (get Facebook account): ___________________

Should you need help to set-up online platforms, we are happy to help you. Please do not hesitate to text or call us in this number (*give your number*). Thank you very much and I am looking forward to your study participation!
